# [Retracted] MicroRNA‑9 inhibits gastric cancer cell proliferation and migration by targeting neuropilin‑1

**DOI:** 10.3892/etm.2024.12687

**Published:** 2024-08-12

**Authors:** Cheng Hang, Hui-Shen Yan, Chen Gong, Hua Gao, Qiu-Hui Mao, Jian-Xin Zhu

Exp Ther Med 18:2524–2530, 2019; DOI: 10.3892/etm.2019.7841

Following the publication of this paper, it was drawn to the Editor’s attention by a concerned reader that certain of the Transwell cell invasion assay data shown in Fig. 3B on p. 3528 and the colony formation assay data in Fig. 4A on p. 3529 were strikingly similar to data appearing in different form in other articles written by different authors at different research institutes that had already been published elsewhere prior to the submission of this paper to *Experimental and Therapeutic Medicine* (one of which has already been retracted). In view of the fact that the abovementioned data had already apparently been published previously, the Editor of *Experimental and Therapeutic Medicine* has decided that this paper should be retracted from the Journal. The authors were asked for an explanation to account for these concerns, but the Editorial Office did not receive a reply. The Editor apologizes to the readership for any inconvenience caused.

